# Application of biological systems and processes employing microbes and algae to Reduce, Recycle, Reuse (3Rs) for the sustainability of circular bioeconomy

**DOI:** 10.3934/microbiol.2022008

**Published:** 2022-03-28

**Authors:** Divakar Dahiya, Hemant Sharma, Arun Kumar Rai, Poonam Singh Nigam

**Affiliations:** 1 Wexham Park Hospital, Wexham Street, Slough Berkshire, SL2 4HL, UK; 2 Department of Botany, Sikkim University, 6th Mile Tadong, Gangtok, Sikkim India; 3 Biomedical Sciences Research Institute, Ulster University, Coleraine, Northern Ireland, UK

**Keywords:** bioeconomy, microorganisms, algae, recycling, reuse, reduce, value-added products, metabolites, enzymes, bioeconomy, sustainability

## Abstract

The circular bioeconomy has undoubtedly gained global momentum during the last few years. The bioeconomy envisions “3R”, the goal of 3R (Reduce, Recycle, Reuse) is to implement in circular economy preventing excessive and unnecessary wastes. The circular bioeconomy emphasizes the best use of all sorts of available bioresources through the reduction of generated wastes during product formation, recycling of generated wastes, and reuse of valuable by-products and residues. Biotechnology could be useful in utilizing the resources to the optimum and therefore the role of biological agents and bioprocesses is of prime importance. In this review, we highlight the paramount importance of beneficial strains of microorganisms, macro, and microalgae in the bioeconomy. Microorganisms are universally recognized for the notable production of a vast array of secondary metabolites and other functionalities with possible use in various sectors. The application of potential strains in industries and modern agriculture practices could progressively improve the effective yield of food and feed, including fertilization of arid soils, bioconversion of by-products from industrial processes, and agriculture wastes. The valuable properties of specifically selected biological agents typically make them suitable candidates for their efficient contribution to circular bioeconomy without hampering the environment.

## 1. Introduction

**Figure 1.**
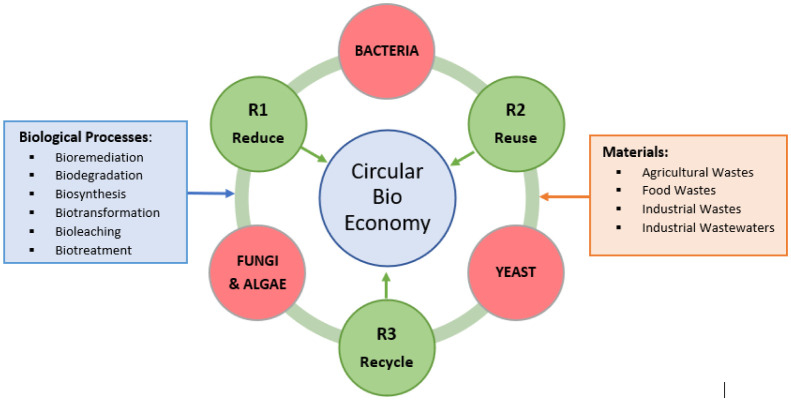
Contribution of biological systems through 3Rs in circular bioeconomy.

Overconsumption of valuable and limited resources has resulted in technological innovation and gradual evolution of innovative business models for considerable ease of successful transition from linear to a circular economy. The marketable products conventionally considered as economic waste in a linear economy can be reprocessed for maximum utilization. A circular bioeconomy typically focuses on the optimal use of bioresources in diverse sectors and extensive use of biotechnological tools for the processing of goods, economic modernization of essential services, and generation of sustainable energy [1]. The role of microorganisms, although limited, is significant in circular bioeconomy for the bioconversion of raw materials, processing of valuable by-products, recycling and decomposition of agriculture, and industrial residual wastes. This review discusses the prospective use of microorganisms in the circular bioeconomy (Figure 1). Extensive studies on microorganisms have amply demonstrated their remarkable ability to produce secondary metabolites, valuable enzymes, plant growth-promoting factors, and an impressive range of desired functionalities. Therefore, selected microorganisms could undoubtedly play a valuable role in the circular economy of different industrial and agriculture sectors. Mainly bacteria, fungi, yeasts, and algae have been employed for the bioconversion of residual wastes and wastewaters generated from agriculture, food, and drink industries. These wastes and residues are rich in organic load, suitable as carbon sources for the cultivation of microbial agents. Figure 1 presents the type of biological processes, used for R1-reduction, R2-reuse, and R3-recycling of materials. Process selection depends on the type of residual material or byproducts that need to be treated, either for their reduction, treatment, or for the bioconversion into value-added products.

### 1.1. Methodology

In this study, we seek to highlight the possible scope of biosystems in the circular bioeconomy, which lacks extensive studies to date. It is important to understand the types of different economies viz. linear economy, circular economy, bioeconomy, and circular-bioeconomy through literature search. Further, the role of microorganisms, micro, and macroalgae in the circular-bioeconomy has been discussed.

### 1.2. Linear economy

Linear economy or conventional system primarily involves the extensive use and improper disposal of materials made by industries, where the raw materials typically enter the system at the start of the complex process, and the by-products are improperly disposed of. The linear economy results in the considerable loss of valuable resources in the production line and eventually adversely affects the environment [2],[3]. The wastage and redundancy of the products at the effective end of their life cycle results in the continuous exhaustion of natural resources [4]. The uncontrolled expansion or diversification of economic activities causes an intense damaging effect on the vulnerable environment, which inevitably leads to disturbance in the economy [5].

### 1.3. Circular economy

The circular economy aims to regenerate and carefully redesign the industrial systems by preserving and enhancing its capital, optimizing the ideal yields, and promoting the overall effectiveness of the system. This can be achieved by several factors:

1. Using renewable sources of energy,

2. By eliminating the use of toxic and hazardous chemicals,

3. Reducing or eliminating generated wastes by utilizing the correct starting raw materials,

4. By implementing innovative process designs and effective models,

5. Recycling and using of wastes generated during the production, or even after the consumption of several types of products [4].

The maximization of the use of the raw materials in the production line and minimization of their loss with time is the basis of a circular economy. Carefully designed models in circular economy help in reducing wastes through personal interaction involving humans, which is important during continuous production and sustainable consumption of products. In comparison to the conventional linear models, the circular economy considers a product as a resource even at the end of its life-cycle, rather than a waste product [6].

The innovative idea of a new product formulation should involve a creative process and methodical approach through which all the wastes are sufficiently reduced at each step. Major principles of circular economy involve recycling of by-products and end-products as well as helping in utilizing resources sensibly and eliminating wastes. Such an approach eventually becomes a beneficial contribution to the global economy [5]. The key difference between a linear and circular model of the economy is that the circular approach is considered a better sustainable system that involves resolving the gaps and disparities related to the limited resources without obstructing the development [7]. Though the model of circular economy has been criticized, by anticipating it to be an unfeasible idea [8]. The objectives of the circular economy could be accepted to encourage all possible productive measures if there is even a slight reduction in the generation of wastes and a drop in the consumption of non-renewable resources [9].

### 1.4. Bioeconomy

There has been considerable interest around the globe for the conversion of the conventional system of utilizing bioresources to sustainable ones. The process generally encompasses the employment of materials derived from biological sources or biomass obtained from wastes generated from different industries [10]. Around 50 countries along with international organizations are framing strategies for the steady transition of the conventional system to bioeconomy through sustainable utilization of biological resources [11]. Bioeconomy accurately represents a multipart mechanism that typically involves numerous sectors with ultimate consumers and cannot be considered merely as an autonomous section of an economy [12].

The bioeconomy aspect involves three main factors [13]:

1. The efficient utilization of renewable biological reserves.

2. Significant transformation of valuable resources.

3. Recycling waste products into beneficial products.

The vision of the bioeconomy has been classified into two main categories by Bugge *et al*. [14] which are:

1. Biotechnology vision in bioeconomy typically assigns significance to Biotechnology research, this involves commercialization and efficient utilization of products derived through the effective use of Biotechnology in various sectors.

2. Upgrading and efficient processing of biological resources, formation of innovative value chains as the prime focus of Bioresource vision of bioeconomy.

The Bioeconomy vision attributes considerable importance to the optimal use of valuable resources, including energy and essential nutrients. Sustainability can be promoted by active upgrading of biological diversity, the apparent reduction in mono-cultures and appreciably reducing the degradation of productive soil. Some of the expected targets of circular economy and bioeconomy are somewhat similar. Both the economies depend on the widespread use of sustainable resources and prevent the use of fossil fuels, which supports to deal with climate change and the greenhouse effect. The circular economy approach typically counts on the extensive use of reprocessed materials and effective systems and technologies, whereas the bioeconomy recommends the large use of energy derived from a natural and renewable source such as agriculture, food industry, forests, and marine biomass [15].

### 1.5. Circular bioeconomy

An effective bioeconomy aims to generate stable and balanced products from available bioresources and its efficient value addition in circular bioeconomy (CBE) [16]. Products with added value are targeted to be generated from bio-resources in a circular bioeconomy [17] that carefully maintains the economic values of resources at their possible optimum use and minimizes the outflow of under-utilized resources by managing the supply of resources satisfactorily [18]. Therefore, the fundamental concept of circular bioeconomy is necessarily based on functional hypotheses of circular economy and bioeconomy intersecting them to significant capacity [15]. This type of Bioeconomy relates to all the production sectors involving bioresources and interlinking with the industrial sectors that typically utilize bioresources for the efficient production of bioproducts to adequately maintain circularity of essential materials for the sustainable use of valuable resources, conserve the pristine environment and carefully maintain the biodiversity [19].

**Figure 2.**
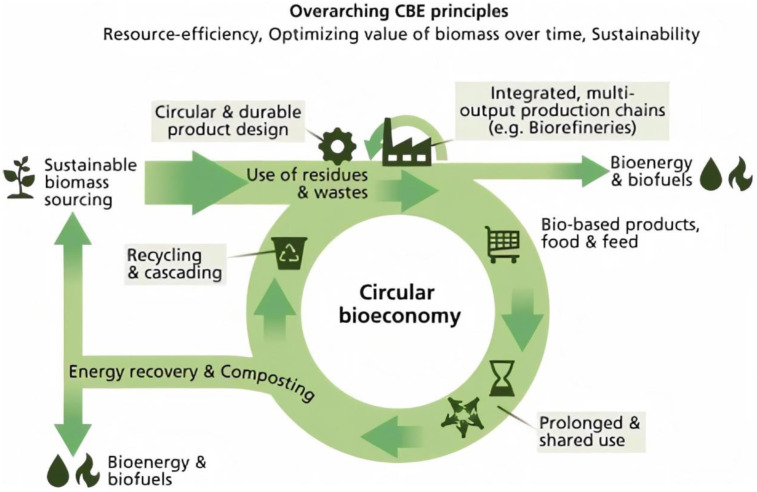
Integrated principles of circular bioeconomy. *Note: This fig was published in Resources, Conservation & Recycling: X, Vol6 by P. Stegmanna, et al, the circular bioeconomy: Its elements and role in European bioeconomy clusters, 100029, Copyright permission to use it in this article has been granted by Senior Copyrights Coordinator, Elsevier on 27th Nov 2021.

The successful development of the bioeconomy currently represents a global trend to ensure paramount safety and adequate access to proper food, raw materials, energy-source, water, and their efficient use. The major principles of circular bioeconomy are shown in Figure 2 [20]. All principles are integrated for the sustainability of the economy, as Stegmanna et al have very well summarized all components overarching the principles of circular bioeconomy in fig 2 [20]. The vital barrier in the implementation of circular economy is the absence of a proper legal framework as studied in the Polish South Baltic Area. One of the important aspects is to simplify the process and administrative approach such as changing the status of waste into raw materials [21].

A circular bioeconomy typically involves treating waste products as valuable resources. Intentionally burning of crop residues is traditionally practiced in some places of the world to prepare the cultivated field for the next continuous cycle of specific crops. This deliberate act of burning of the residues results in an adverse effect on human health and the environment along with a considerable economic loss to the prospective farmers. Instead, biochar produced from wheat-straw along with specific nitrification-inhibitor sourced from neem plants with the recommended dose of synthetic fertilizer could significantly improve soil nutrition for good maize crops. This highlights the vital importance of agriculture waste as a valuable resource in the circular bioeconomy [22].

## 2. Significant application of biological systems

**Figure 3.**
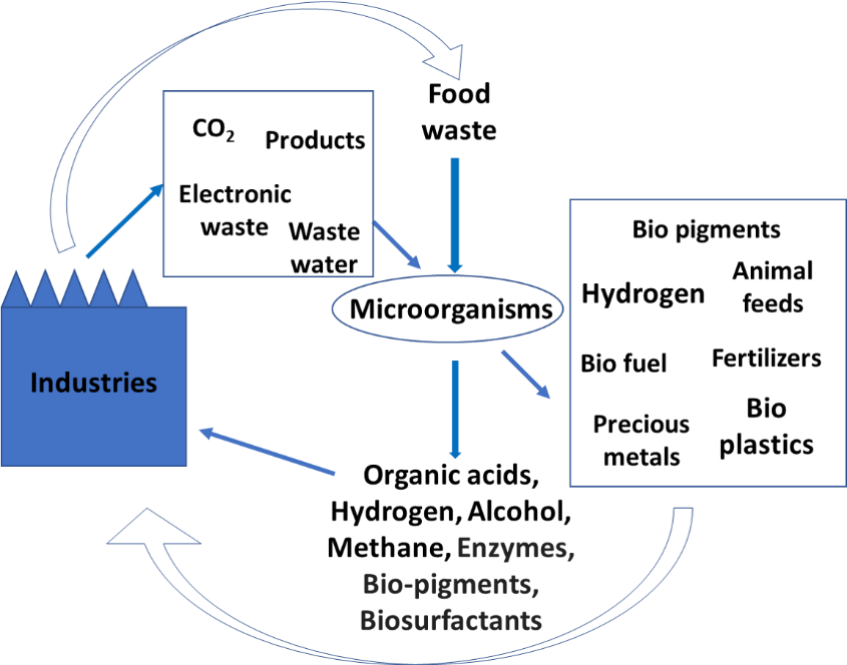
Role of microorganisms in the recycling of materials in the industrial sector.

Bioprocesses employing microorganisms could help in the recycling of industrial wastes and play an important role in the circular bioeconomy. There are several projects undertaken globally by researchers for such studies. Figure 3 presents the information on the role of microorganisms in the synthesis of a diverse range of metabolites, including biofuels, feeds, enzymes, pigments, organic acid, and other compounds of economical value. Several of these compounds are either used as an energy source and/or raw materials for the synthesis of other added-value products in industries.

### 2.1. Bioleaching

Structural modeling was conducted to assess the use of microorganisms for the extraction of metals from electronic wastes through biotechnology. Bioleaching of metals employing microorganisms is an eco-friendly approach in the extraction of metals from electronic waste. A bacterial species belonging to the *Paenibacillus* genera was found to be suitable for bioleaching copper, cadmium, sodium, and lead from electronic wastes viz. video card and random-access memory card [23]. Successful extraction of multiple metals from the closed-circuit board was carried out using *Acidithiobacillus ferrooxidans* [24]. Single-stage or double-stage bio-hydrometallurgy processes are used for the extraction of metals using different types of microorganisms, such as *Sulfobacillus thermosulfidooxidans, Aspergillus niger*, *Penicillium simplicissimum, A. thiooxidans*, and *A. ferroxidans*. Such approaches were applied through several mechanisms, such as acidolysis, redox reactions, bioaccumulation, and the complexation of metals [25]. It has also been stated that the activities of microorganisms could be able to lessen the release of enormous amounts of CO_2_ and save water resources [26].

### 2.2. Biosynthesis of molecules of added value

Biomolecules produced by cyanobacteria and microalgae could be used as material for the synthesis of bioplastics. The methane generated during the process could be used as a precursor molecule for resynthesis [27]. *Synechocystis salina* was employed for the biosynthesis of poly-hydroxybutyrate, along with the production of useful by-products, such as feeds for animals, bio-pigments, biomethane, and fertilizers. Such a process contributes to decreasing the carbon footprint in the environment [28]. Valorization of food wastes, which involves increasing the value of the waste products, was possible for the production of economically-valuable products, such as lactic acid, plasticizer, human and animal feed through biotechnology. In the past few decades, more than 75% of the annual microalgae biomass has been used by the health and food market for the formulation of powders, tablets, and capsules. Spirulina, a filamentous blue-green alga is used worldwide as a food supplement in the form of tablets, flakes, or powder [17],[29]. The biosynthesis of microbial lipids has been used in biofuel production as currently biodiesel is an alternative to diesel fuel used in transportation. For this purpose, microalgae are specifically selected and being cultivated for their unique characteristics of fast-growing microorganisms. Microalgae double in their biomass within 24 hours under daylight conditions, which is up to 5-fold higher mass productivity as compared to traditional oilseed-producing crops. The harvested biomass of some strains of microalgae may contain more than 80%,w/w lipids on a dry biomass weight basis, which is 7 to 31% higher oil yields of microalgae as compared to palm oil if employing selected strains of microalgae such as *Chlorella pyrenoidosa* [29]. Further details on lipid biosynthesis and its applications have been discussed in sections 3.2 and 3.3 including more references from published research. A photo-fermentation process employed *Rhodobacter sphaeroides* B-3059 to achieve another type of biofuel, the hydrogen, bioconverting the valuable organic load present in the distillery wastewater [30].

Electrosynthesis using specific microorganisms involves the use of microbial cells to accept electrons and reduce carbon dioxide that could be useful for recycling CO_2_ into valuable by-products, which provides an insight into the extensive use of microorganisms in circular bioeconomy [31]. Lin *et al* [32] have reported the possible use of *Actinobacillus succinogenes* for biofuel after evaluation of thermal characteristics of the bacterium. With a higher combustibility index than lignite coal and almost similar to biochar and bio-oil, the bacterium could be developed as fuel and play a part in the bioeconomy. Thus, specialized microorganisms could be used for biobased products from the sequestration of CO_2_, to ease climate change along with the production of value-added products [33]. Quite a number of microorganisms are well known for their capability to secrete bioactive molecules with therapeutic properties including, antimicrobials [34], antitumor agents [35], antiviral [36], anti-glycemic [37], antitubercular [38], anti-plasmodial [39], cytotoxic [40], anti-inflammatory [41] and cholesterol-lowering agents [42].

### 2.3. Bioprocesses employing microorganisms in organic farming-practice

Some of the key characteristics of microorganisms make them ideal candidates for use in organic farming practice. A diverse number of microorganisms have been isolated that display antimicrobial potential against phytopathogens and some produce molecules with bio fertilization properties [43]–[45]. Microorganisms secreting antimicrobial metabolites certainly help the vulnerable plants to persistently resist infections from phytopathogens. With enhanced support from these beneficial microorganisms, plants have coped better under unfavorable and hostile conditions [46]–[48]. Some of the microbial species can secrete phosphate solubilizing enzymes that effectively provide plants with phosphates from the soil [49]. Phosphate solubilizing microorganisms release organic acids into the soil that solubilize inorganic phosphate complexes into ortho-phosphates and make them available for utilization by the plants [50].

Microorganisms secrete siderophores that chelate iron molecules present in the surroundings and thus severely inhibit the continuous growth of pathogens by limiting the essential nutrients. Some of the microorganisms promote plant growth in several ways, supplying chelated or sequestered iron to the host plant [51],[52]; producing plant hormones indoleacetic acid [53] and gibberellins [51],[54], and by fixing atmospheric nitrogen [55]. Although, few microorganisms such as *Neotyphodium lolii* may cause a negative effect on plant growth and induce dwarfism in *Lolium perenne* [56]*,* the potential of beneficial microorganisms, such as endophytes in plant growth promotion outranks its adverse effects on plants. In a study conducted to assess the effect of grazing on non-toxic tall fescue infected with novel endophyte, improvement in the rate of calving, production of milk, and growth of calf were observed [57].

**Table 1.** Metabolites and functionalities of microorganisms in pharmaceutical, agriculture, and industrial sectors.

Sl. No.	Microbial parts/products	Microorganism	Activity	Reference
1	Pestalotiopisorin B	*Pestalotiopsis* sp.	Antibacterial	[70]
2	Oxysporone	*Pestalotia* sp.	Antibacterial against MRSA strains.	[71]
3	Xylitol	*Pestalotia* sp.	Antibacterial against MRSA strains	[71]
4	Desmethyldichloro-diaportintone	Ascomycota CYSK-4	Anti-inflammatory	[72]
5	Serine glycine betaine	*Macrophomina phaseolina*	Anti-cancer	[73]
6	1,3,5,6-tetrahydroxy-8-methylxanthone and 1,6-dihydroxy-3-methoxy-8-methylxanthone.	*Penicillium canescens*	α-glucosidase inhibitors	[37]
7	IAA, Ammonia, and HCN production.	*Bacillus altitudinis* GTS-16	Plant growth promotion and induction of systemic resistance against *Rhizoctonia solani* in rice	[74]
8	Phosphate solubilization, Siderophores production, and Insecticidal properties.	*Beauveria bassiana*	Growth promotion of tomato plants and inhibition of *Trialeurodes vaporariorum*	[75]
9	Amylase, protease, cellulase, pectinase, and lipase.	*Pseudopestalotiopsis theae*	Enzymes of industrial importance	[76]
10	Mycelium	*Rhizophagus intraradices*	Stimulate NH_4_ absorption by plants and improve nitrogen use efficiency.	[77]
11	Biomass	*Actinobacillus succinogenes*	Microorganisms as fuel	[32]
12	Biodegradable polymer	*Aspergillus* sp., *Penicillium* sp., *Fusarium* sp.	non-toxic, biodegradable, and biocompatible products	[78]

### 2.4. Microbial assisted treatment of pollutants

Pollution of valuable fertile soil has become a common careless practice in many countries. Some synthetic or natural compounds are present in the contaminants, which require efficient approaches for their removal. Biotreatment of such pollutants through the strategy of microbial-assisted remediation is one of the effective and cost-effective methods. An array of mechanisms is involved in microbial remediation of pollutants *viz*. absorption, uptake and accumulation of metals, precipitation of metals outside the cells, oxidation, and reduction of pollutants through enzymatic activity [58]. Some of the microorganisms isolated from plants play an important role in the remediation of contaminated soils [59],[60].

## 3. Contribution of biotechnology in circular bioeconomy

Table 1 shows summarized information on the possible contribution of microorganisms in the bioeconomy, producing compounds of added value. Certain microorganisms can be used in *in vitro*-biosynthesis of enzymes, which are of importance for their application in several industries, such as amylase [61], protease [62], lipase [63], pectinase [64], cellulase [65], and xylanase [66]. Other value-added biomolecules of economic importance produced by selected strains of microorganisms include bio-pigments [67], biofuels [68], and bio-degraded complex material that have a wide range of uses in the industrial sector [69], having a role in boosting bioeconomy.

### 3.1. Role of microorganisms

There is a possibility of isolating a considerable number of beneficial microorganisms from this habitable planet. Some of the strains have scientifically proven beneficial due to their capabilities to biosynthesize a range of bioactive compounds, which are secreted in their stationary growth phase as secondary metabolites with their possible use in various industrial processes. Effective use of microorganisms could prove beneficial in the synthesis of those molecules, which were traditionally derived from other expensive and non-sustainable sources [79],[80]. Endophytes could be reliably used for the promotion of plant growth under hostile conditions, improving feeds for animals, playing an active role in plant defenses, and increasing the nutrient content of arid soil [79]. This approach will sufficiently reduce our direct dependence on valuable plants for their active metabolites and will result in intelligent environment-friendly uses of plant-based resources.

Microorganisms could efficiently perform a significant role in the bioprocessing of valuable materials in modern industries. Mining novel biocatalysts isolated from exotic habitats could prove useful at diverse levels of cascading processes in key industries. The diverse biotechnological approaches that could be profitably employed to harness the optimum potential include i) Meta-transcriptomics, Meta-proteomics, and Metabolomics; ii) Profiling of crude samples from the diversified environment through whole-genome sequencing techniques; iii) Possible reconstruction of metabolic pathways [81]. Secondary metabolites secreted by microorganisms are far lesser than the ones estimated through genome mining, which could be due to the inactivation of silent biosynthetic gene clusters under *in vitro* studies. Modern genome mining and metabolomics approach using biotechnological tools could be properly employed to mine for such specific genes and sequencing could positively enhance the characterization process [79].

Microbial processes could be a valuable strategy in circular bioeconomy with their extensive application: 1. at the start of the complex process for efficiently generating specific products, 2. during the process, and 3. after the end of products lifecycle by naturally decomposing the used products/byproducts into environment-friendly components.

### 3.2. Role of macroalgae

The development of biorefineries based on the accepted varieties of macroalgal strains have shown the prospects of generating novel products [82]. Several biochemical constituents from macroalgae are known for their industrial value. The generation of biobased products has significant potential for their commercialization which contributes to the economy. Polysaccharides extracted from macroalgae have hydro colloidal and stabilizing properties, which are used in the food and textile industries [83]. Macroalgae have a broad range of biological activities, which have contributed to the circular economy through their application in pharmaceutical, medical, therapeutic, nano-medical, and biological industries [84],[85]. Researches have proved the beneficial activity in macroalgae biomass as anti-oxidant [86], anti-inflammatory [87], anti-coagulant [88], anti-cancer activity [89], and anti-biotic properties in their extracts [90]. The successful application of macroalgal cultivation on an industrial scale is useful for the extraction of several useful polysaccharides like alginate, agarose, carrageenan, and ulvan, which are commercially used in several research studies for media preparation, as well as in food and drink products. Such thickening and gelling agents derived from algal sources are cheaper and provide added value to several products and services [91]–[95].

### 3.3. Role of microalgae

The application of microalgae for a wide range of products has proved it as a sustainable renewable bioresource support system for circular bioeconomy [96]. Microalgae through the bio-fixation of atmospheric carbon dioxide and assimilation of nutrients available in wastewater generated from food industries contribute to circular bioeconomy. As a valuable resource microalgae have been studied widely for the production of renewable energy sources of fuels, like biodiesel [97],[98]. In this way, there is increased use of microalgal biomass, with the bioremediation of organic loads in wastes, and at the same time reducing the environmental pollution caused due to the inadequate disposal of organic residues in streams of wastewater [99],[100]. Besides, integrated processes in microalgae biorefineries with a circular bioeconomy approach not only increase the recovery of resources, but also the efficiency and the profitability of the process. Commercial-scale cultivation of microalgae for the treatment of industrial wastes and the use of harvested algal biomass for biofuel production contributes to circular bioeconomy [101].

Microalgal strains *Aphanizomenon flos-aquae, Arthrospira platensis, Chlorella luteoviridis, Chlorella pyrenoidosa, Chlorella vulgaris, Tetraselmis chuii*, and *Odontella aurita*, have been included in the list of foods and ingredients authorized in the European Union. Several bioactive compounds produced by microalgae have been approved as food ingredients by EFSA, some are biopigments like β-carotene from *Dunaliella*, phycocyanin from *A. platensis*, Docosa-hexaenoic acid from *Crypthecodinium cohnii*, and Astaxanthin from *T. chuii* and *Haematococcus* [96]. Micro and macroalgal species have been reported as soil improvers and sources of necessary nutrients for crop production in experimental greenhouses as well as their application in actual field conditions [102]. A novel microalgal species of Chlorella has been reported for the production of microalgal biomass and lipid synthesis utilizing dairy industry effluent [103].

The manufacturers of aquafeed have been successful in reducing the contents of fishmeal and fish oil by cost-competitive replacements. Two commercially available microalgae have been studied to produce a high-performing fish-free feed for the world's second-largest group of farmed fish *Oreochromis niloticus*. Researchers substituted fishmeal with a protein-rich defatted biomass of *Nannochloropsis oculata*, which was available as a useful leftover after the extraction of oil for nutraceuticals); whereas the fish oil could be substituted using whole cells of *Schizochytrium* sp. as a source of docosahexaenoic acid [104]. This work is a useful contribution to the circular economy by eliminating the dependency on fishmeal and fish oil, which produced a cost-effective feed product. This microalgae-based feed has a better commercial value with improved growth metrics and the nutritional quality of the farmed fish. This seems to be a preferred option using microalgal-based feed over insect-meal-based supplements for fish feed [105].

Microorganisms are economical agents contributing to circular bioeconomy, though their activities can be utilized through several routes as discussed in previous sections, however, the biosynthesis of polyunsaturated fatty acids (PUFA) is another economical proposition. The organic residues and wastes generated in many industries are valuable carbon-load-containing resources to be used as the growth media for the cultivation of PUFA producing microorganisms [106]. Several strains of oleaginous microorganisms, including algae and fungi, have been studied for their capability of biosynthesizing lipids known as single cell oils (SCOs) containing PUFAs. The exploitation of these organisms on a commercial scale at lower-fermentation cost can be achieved, if two processes are combined - the biosynthesis of SCO in the fermentation process, and the valorization of residual wastes and by-products of industries such as distilleries, sugar, food, and agriculture [97]. Microorganism belonging to the class of Mucoromycota, Thraustochytrids (fungoid-like), GMO-*Yarrowia lipolytica*, and microalgae Isochrysis, Nannochloropsis, and Tetraselmis have shown their ability to produce PUFAs. Among types of PUFA, omega-3 (ALA, 18:3n-3) and omega-6 (LA, 18:2n-6) are two essential fatty acids for human health [107],[108], and also reported as essential for external administration to prevent certain health complications [109]. The other two main advantages of PUFA production employing microbial agents, apart from the valorization of wastes, are non-dependency of production process on climatic conditions and no requirement of arable land. Such an approach for PUFA biosynthesis under laboratory conditions for the production of value-added products like biodiesel and dietary supplements causes no concern for the negative effect on the ecosystem [110]–[111], which is another contribution toward the sustainability of the economy.

## 4. Knowledge gaps

There has been considerable progress in the exploration of microorganisms isolated from various habitats across the globe for their employment in bioprocesses. However, a large part of the planet remains unexplored to date. Investigation of microorganisms from deserts, alpine regions, mangrove forests, flooded grasslands, etc. may reveal the array of novel functionalities [112],[113]. There is less understanding of mechanisms of microorganisms' interactions with the crop plants. Some of the microorganisms including endophytes have proven to be beneficial in promoting plant growth. However, their interaction with the native microorganisms residing in the host plants requires further investigation [114]. Further research is required to explore their probable and effective application in the industrial bioprocesses for the utilization of a variety of residual materials, which are generated annually as bioresources in several sectors globally [115]–[118] to contribute to circular bioeconomy on a commercial scale [119]–[120]

## 5. Conclusion

The conventional economic system that typically involves the key concept of use and throw of products undoubtedly puts considerable pressure on valuable resources and in turn, it causes a detrimental effect on our precious environment. The circular economy expects the adaptive reuse of valuable resources and it progressively reduces wastes through well-designed strategies. The bioeconomy naturally implies the extensive use of renewable sources of energy and relies on the proper use of natural substances as raw materials. The circular bioeconomy adjoins the circular economy and bioeconomy together. However, accurate identification of fortes and potential weakness at the regional level is a must for a favorable and successful transition.

The principal importance of beneficial microorganisms, micro and macroalgal species in the circular bioeconomy has been highlighted in this short review. Biological agents have the ability to recycle and positively transform an impressive array of valuable materials including wastes produced from various modern industries. The microorganisms are laden with immense potential, which has remained mostly unused to date in this sector. Biotechnological tools could undoubtedly enhance their unique abilities to improve the continuous production of food and feeds. Through proper recycling and reuse of agriculture and food wastes, a range of bioactive metabolites and industrially significant enzymes can be produced. The renewable raw materials and industrial wastes can be transformed for the maximum utilization of limited resources while employing a greener approach towards the circular bioeconomy. The active role of biological systems is of prime importance in circular bioeconomy through widening the application spectra of the beneficial microorganisms, macro, and microalgal species.
